# The conserved RNA recognition motif and C3H1 domain of the Not4 ubiquitin ligase regulate *in vivo* ligase function

**DOI:** 10.1038/s41598-018-26576-1

**Published:** 2018-05-25

**Authors:** Hongfeng Chen, Tirupataiah Sirupangi, Zhao-Hui Wu, Daniel L. Johnson, R. Nicholas Laribee

**Affiliations:** 1 Department of Pathology and Laboratory Medicine, and Center for Cancer Research, University of Tennessee Health Science Center, Memphis, TN 38163 United States of America; 2 Molecular Bioinformatics Core and the University of Tennessee Health Science Center Office of Research, University of Tennessee Health Science Center, Memphis, TN 38163 United States of America

## Abstract

The Ccr4-Not complex controls RNA polymerase II (Pol II) dependent gene expression and proteasome function. The Not4 ubiquitin ligase is a Ccr4-Not subunit that has both a RING domain and a conserved RNA recognition motif and C3H1 domain (referred to as the RRM-C domain) with unknown function. We demonstrate that while individual Not4 RING or RRM-C mutants fail to replicate the proteasomal defects found in Not4 deficient cells, mutation of both exhibits a Not4 loss of function phenotype. Transcriptome analysis revealed that the Not4 RRM-C affects a specific subset of Pol II-regulated genes, including those involved in transcription elongation, cyclin-dependent kinase regulated nutrient responses, and ribosomal biogenesis. The Not4 RING, RRM-C, or RING/RRM-C mutations cause a generalized increase in Pol II binding at a subset of these genes, yet their impact on gene expression does not always correlate with Pol II recruitment which suggests Not4 regulates their expression through additional mechanisms. Intriguingly, we find that while the Not4 RRM-C is dispensable for Ccr4-Not association with RNA Pol II, the Not4 RING domain is required for these interactions. Collectively, these data elucidate previously unknown roles for the conserved Not4 RRM-C and RING domains in regulating Ccr4-Not dependent functions *in vivo*.

## Introduction

Gene expression is dynamically regulated by the environment and is controlled at multiple stages for cells to maintain homeostasis. While studies of the RNA polymerase II (Pol II) transcription cycle subdivide this process into the initiation, elongation, and termination phases, in reality these processes function in a continuum that involves a suite of multifunctional Pol II co-regulators^1^. The eukaryotic Ccr4-Not complex functions at every stage of the mRNA lifecycle, from controlling Pol II initiation and elongation, to participating in mRNA nuclear export, translational regulation, and mRNA decay^2,3^. Originally identified in budding yeast, the nine core subunits (Not1-Not5, Ccr4, Caf1, Caf40, and Caf130) form a stable macromolecular complex of approximately 1.0 MDa in size. The complex is conserved in metazoans where it contains additional subunits not found in yeast^4^. The essential Not1 subunit serves as a scaffold upon which the other subunits assemble to form a modular complex^5–8^. Ccr4-Not functions predominantly as a holocomplex, although sub-complexes may form *in vivo* to regulate a subset of its functions. Because it controls all stages of Pol II-dependent gene expression, as well as Pol I transcription^9^, Ccr4-Not may integrate environmental information to coordinate gene expression mediated by different transcription systems^10^.

Integral to yeast Ccr4-Not are the enzymatic activities provided by the Ccr4 and Not4 subunits. Ccr4 is an RNase belonging to the exonuclease-endonuclease-phosphatase (EEP) enzyme family that specifically degrades polyadenylated mRNA and regulates global mRNA decay^2,11–13^. Caf1 is an RNase belonging to the DEDD superfamily. While yeast Caf1 lacks conserved residues in the active site required for catalysis and is not an active enzyme, its metazoan orthologs contribute substantially to mRNA turnover *in vivo*^12,14–16^. In contrast, the unique Not4 ubiquitin ligase remains poorly characterized in both yeast and mammals. Not4 contains an N-terminal C_4_C_4_ RING ubiquitin ligase domain that interacts with the Ubc4 and Ubc5 (Ubc4/5) E2 ubiquitin conjugating enzymes to mediate substrate ubiquitylation^17–19^. The yeast Not4 C-terminus, which lacks identifiable domains, is responsible for stable integration into Ccr4-Not^20^. This region is less conserved with metazoan Not4; consequently, metazoan Not4 does not stably integrate into the core Ccr4-Not complex^6,21^. Adjacent to the RING domain is an RNA recognition motif (RRM) and a C3H1 domain, which we collectively denote as the RRM-C domain, whose functions are not defined^22,23^. Intriguingly, how Not4 recognizes its few defined substrates is unclear, suggesting the possibility that the Not4 RRM-C domain may play a role. The RING and RRM-C domains are highly conserved throughout evolution such that human CNOT4 can complement a *not4Δ* mutant^17^. Although few Not4 substrates are known, yeast lacking Not4 exhibit substantial growth impairment and wide-ranging sensitivity to many environmental stressors thus illustrating its importance *in vivo*^18,24^. One well characterized Not4 substrate is Jhd2 which is the sole histone H3 lysine 4 trimethylation (H3K4me3) demethylase in yeast. Not4 loss causes a global decrease in H3K4me3 due to increased Jhd2 stability^25–27^. Other known Not4 substrates include translational quality control components, the stress transcription factor Yap1, and the cyclin C subunit of the Mediator complex^28–30^. Yet none of these substrates explain the importance of Not4, thus suggesting additional substrates remain to be identified.

Ccr4-Not positively and negatively regulates Pol I and Pol II transcription through unclear mechanisms. Whether the Not4 RRM-C contributes to transcriptional control or mRNA turnover by binding RNA remains unknown, yet Ccr4-Not association with Pol II is thought to be independent of RNA binding^31^. Ccr4-Not also controls global proteostasis through Not4-dependent regulation of proteasome assembly and activity. In *not4Δ* cells, proteasomes form aberrant, salt-resistant structures exhibiting defects in 19S regulatory particle (19S RP)-dependent substrate deubiquitylation, while 20S core particles (20S CP) have increased *in vitro* catalytic activity^20,32^. How Not4 controls proteasome activity is unclear, although part of its role involves regulating the stability of the proteasome associated factor Ecm29 which participates in proteasome assembly and is a proteasome negative regulator^20,33^.

The combination of RRM and C3H1 domains makes Not4 unique among eukaryotic E3 ubiquitin ligases^34^. To define if the RRM-C domain contributes to Not4 function *in vivo*, we generated mutations at highly conserved residues within the RRM-C and assessed their contribution to Not4-dependent proteasome regulation and Pol II transcription. We find the Not4 RRM-C collaborates with the RING domain to control proteasome activity and global proteostasis. Transcriptome (RNA-seq) analysis reveals the RRM-C selectively affects Pol II-dependent gene expression involved in biological pathways related to transcription elongation, cyclin-dependent kinase (CDK) dependent nutrient responses, and ribosome biogenesis. In addition, while the RRM-C domain does not affect Ccr4-Not association with Pol II, we demonstrate that the Not4 RING domain is essential for these interactions. Our study thus provides the first evidence that the evolutionarily conserved Not4 RRM-C domain is required for *in vivo* Not4 function.

## Results

### Generation and *in vivo* characterization of the Not4 RRM-C mutant

**Figure 1:**
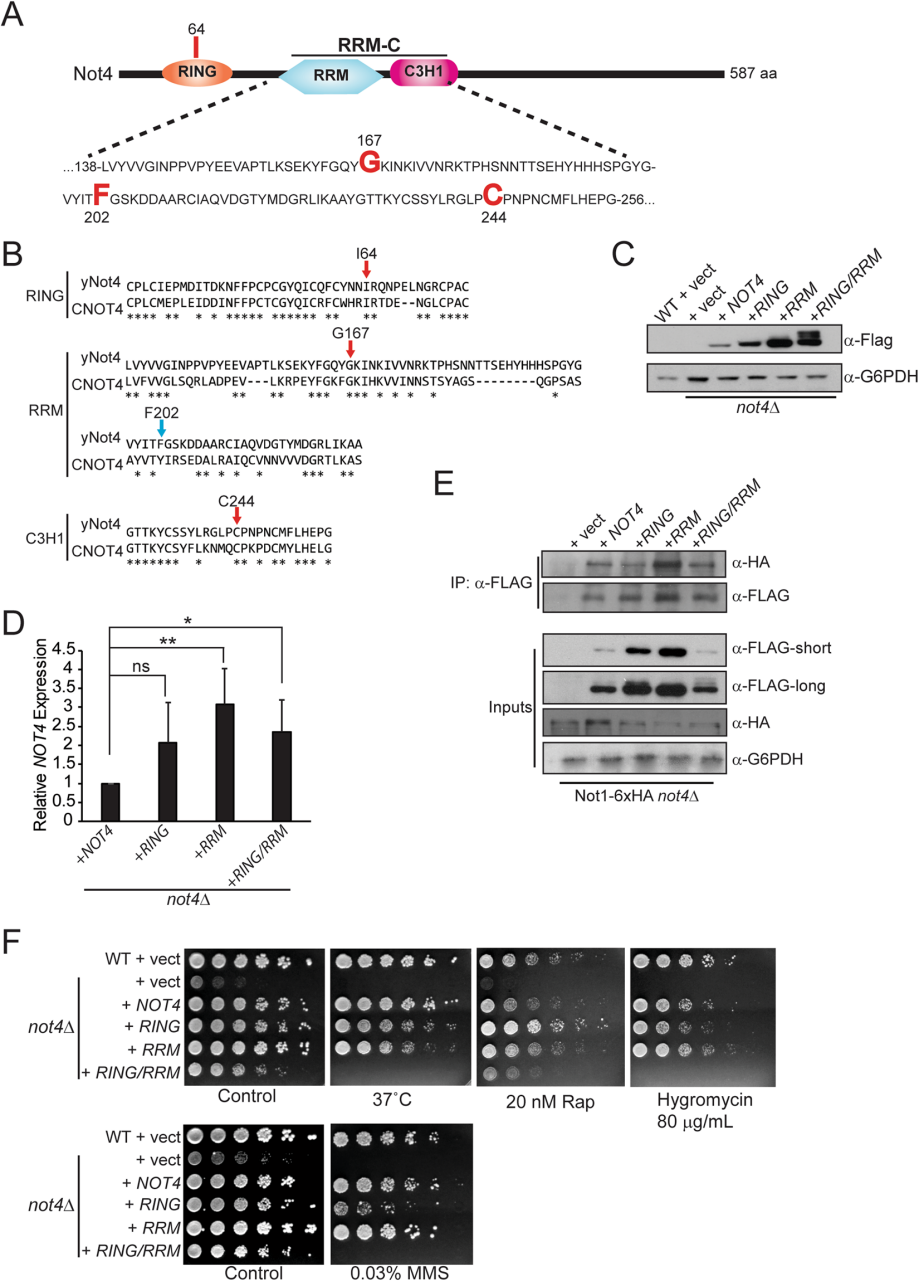
The Not4 RRM and C3H1 domains contribute to Not4 function *in vivo*. (**A**) Schematic of the domain architecture of Not4. Sequence of the RRM and C3H1 domains is shown. The residues mutated in the RING, RRM and C3H1 domains are indicated in large, bold red letters. The RRM and C3H1 combinatorial mutant is referred to throughout as the Not4RRM mutant. (**B**) Amino acid alignment of the RING, RRM, and C3H1 domains from yeast Not4 (yNot4) and human CNOT4. The red arrows indicate invariant residues while the blue arrow indicates a conservative difference (phenylalanine to tyrosine). (**C**) Immunoblot of log phase cell extracts from wild-type (WT) cells transformed with control vector, *not4Δ* transformed with control vector, or *not4Δ* transformed with vectors expressing C-terminal FLAG tagged Not4 WT (*NOT4*) or the indicated Not4 mutants. (**D**) The *not4Δ* cells expressing Not4WT, Not4RING, Not4RRM, Not4RING/RRM were subjected to RT-qPCR analysis to quantify *NOT4* mRNA expression. The data are the average and SD of five independent experiments with significance determined by two-sided Student’s t-test. *p < 0.05; **p < *0.01*. (**E**) Not1-6XHA *not4Δ* cells were transformed with the indicated Not4 expression vectors and the association of Not4WT and the different Not4 mutants with Not1 was assessed by α-FLAG IP and α-HA immunoblot. (**F**) The indicated strains were cultured overnight and then equal numbers of cells were 5-fold serially diluted and spotted to plates containing control media or media with the indicated agents. All plates were incubated at either 30 °C or 37 °C for 4–6 days.

The Not4 RING residues necessary for Ubc4/5 interaction were defined previously, while the Not4 C-terminus was shown to mediate stable Not4 integration into Ccr4-Not^18,20^. Yet a functional role for the highly conserved RRM-C domain (shown in Fig. 1A and aligned against human CNOT4 in Fig. 1B) was never determined within the context of full-length Not4. To address the role of the RRM-C, we utilized the analysis tools available in the SMART and PROSITE databases to identify conserved residues shared with other RRM domain factors^35–38^. We identified both glycine 167 (G167) and phenylalanine 202 (F202) to be highly conserved amongst many RRM containing proteins, as well as a key conserved cysteine residue (C244) in the C3H1 domain. These residues were changed to alanine to generate the triple mutant (G167A F202A C244A), hereafter referred to as Not4RRM (Fig. 1A). Wild-type and *not4Δ* cells were transformed with control vector, or vectors expressing C-terminal FLAG-tagged wild-type Not4 (Not4WT), the previously characterized Not4 I64A which disrupts interaction with E2 enzymes (referred to hereafter as Not4RING) (Fig. 1A,B)^18^, Not4RRM, or the Not4 RING + RRM (Not4RING/RRM) mutants, and their protein expression was assessed by α-FLAG immunoblotting. The WT and different mutants were readily detectable, with higher expression observed for the Not4RRM and Not4RING/RRM mutants relative to Not4WT, while the Not4RING expressed at an intermediate level (Fig. 1C). Interestingly, the Not4RING/RRM exhibited a slower migrating form relative to Not4WT, suggesting this mutant may have differences in its modification status since Not4 is post-translationally modified^17,39^. Reverse transcription coupled with qPCR (RT-qPCR) analysis revealed increased *NOT4* mRNA in Not4RRM and Not4RING/RRM cells relative to Not4WT (Fig. 1D). These data demonstrate that the increased protein expression in these mutants is due at least in part to elevated mRNA levels.

To confirm the different Not4 mutants incorporate into Ccr4-Not, vectors expressing Not4WT and the individual mutants were transformed into *not4Δ* cells expressing an integrated C-terminal 6XHA epitope tag at the *NOT1* genomic locus. Cell extracts from log phase cells were prepared and immunoprecipitated with α-FLAG antibody. Both Not4WT and the Not4 mutants readily co-precipitated Not1 (Fig. 1E), thus demonstrating they all associate with Ccr4-Not. We do note that, for reasons not currently understood, we consistently detect less Not4RING/RRM expression in cells also expressing Not1-6XHA; however, the reduced expression of Not4RING/RRM does not prevent its integration into Ccr4-Not (Fig. 1E). Next, we assessed the ability of Not4WT and the various mutants to rescue a diverse set of *not4Δ* phenotypes. Wild-type (WT) and *not4Δ* cells expressing control vector, or the different Not4 constructs, were cultured overnight to saturation. Equal numbers of cells then were five-fold serially diluted and spotted to control media, or media containing the indicated concentrations of the specific target of rapamycin complex 1 (TORC1) inhibitor rapamycin, the translation inhibitor hygromycin B, or the DNA damaging agent methylmethane sulfonate (MMS). In addition, cells were spotted to duplicate control plates and incubated at 37 °C since *not4Δ* is temperature-sensitive^18,24^. On control plates, and in the presence of the different stressors, *not4Δ* with empty vector grew either poorly or not at all, whereas Not4WT grew under all conditions tested (Fig. 1F). The Not4RING mutant, which prevents interaction with its partner E2 enzymes to impair Not4 ligase activity^18^, exhibited only minor sensitivity to MMS and was not substantially sensitive to the other stressors (Fig. 1F). These results demonstrate that disruption of Not4 RING function alone is insufficient to phenocopy a *not4Δ*. The Not4RRM mutant also grew similarly to Not4WT under all the conditions examined, while the Not4RING/RRM mutant failed to grow at 37 °C, in the presence of hygromycin or MMS, and it only weakly grew in the presence of rapamycin (Fig. 1F). Therefore, *in vivo* Not4 requires both the RING and RRM-C domains to mediate its functions within Ccr4-Not.

### Not4 requires both the RING and RRM-C domains to maintain proteostasis

**Figure 2:**
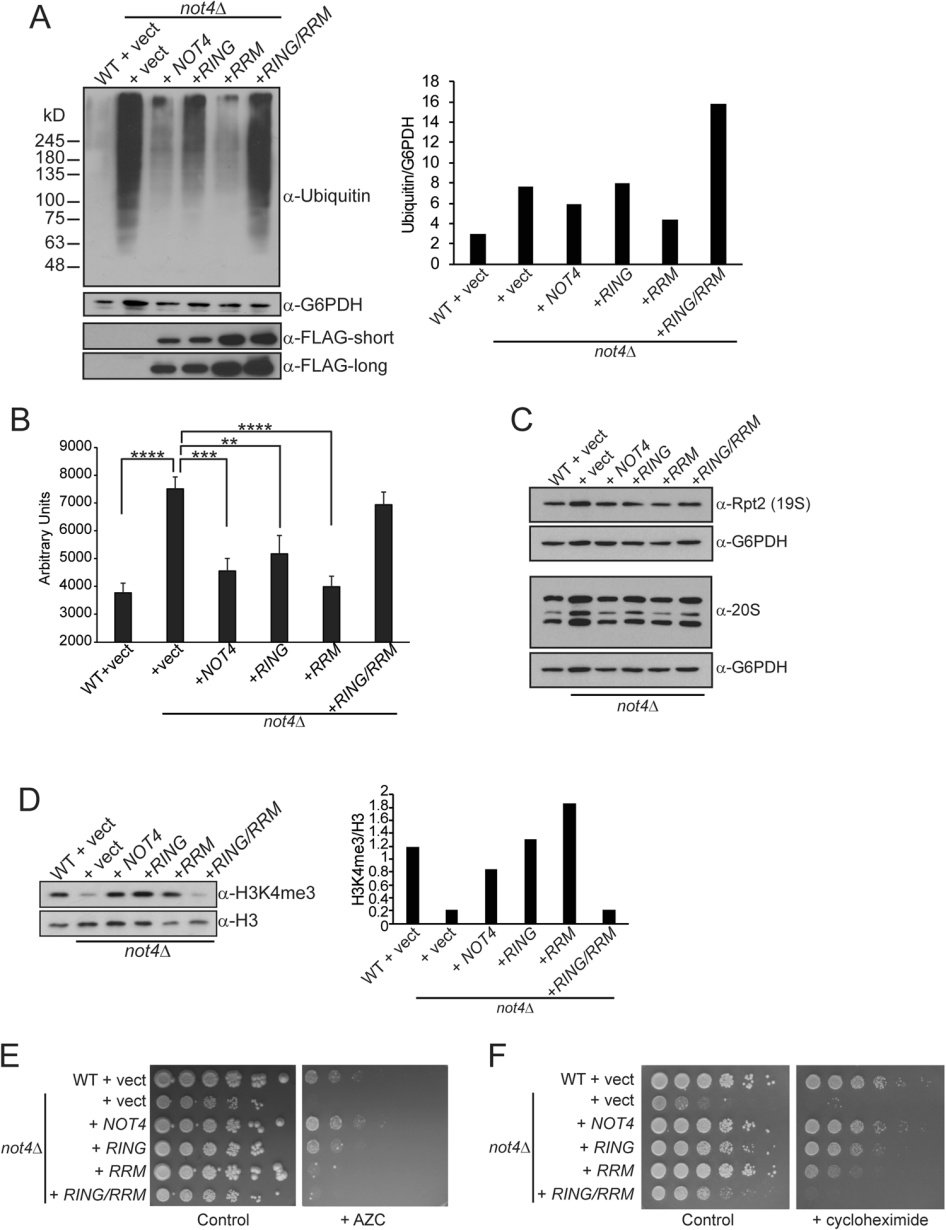
Not4 proteostasis regulation requires the RRM-C domain. (**A**) Log phase extracts were prepared from the indicated strains under denaturing conditions, extracts were resolved by 7.5% SDS-PAGE, and then immunoblotted with α-ubiquitin. Blots were stripped and re-probed with α-FLAG and α-G6PDH to control for loading, and the total ubiquitin signal was quantified and normalized to G6PDH levels. Data are representative of four independent experiments. Note that because *not4Δ* control vector cells consistently express higher amounts of G6PDH, the quantified results underestimate the true increase in global polyubiquitylation in this sample. (**B**) Cell extracts from the indicated strains were prepared, incubated with the LLVY-AMC fluorescent substrate, and fluorescence quantified using a fluorescent plate reader. Triplicate independent extracts were analyzed per sample and the average and SD are plotted. Statistical significance was determined by pairwise (relative to Not4WT) two-sided Student’s t-test. **p < 0.01; ***p < 0.005; ****p < *0.001*. (**C**) Immunoblot of extracts from (**B**) using α-Rpt2 (a 19S proteasome subunit) or α-20S. Note that the α-20S antibody recognizes multiple 20S subunits that have nearly identical mass so their signals overlap. (**D**) Immunoblot analysis of H3K4me3 and total H3 from the indicated strains. The H3K4me3 and H3 signals were quantified and expressed as a ratio. Data are representative of a minimum of four independent experiments. (**E**) Equal numbers of cells were 5-fold serially diluted and spotted to control media or media containing 0.5 mg/mL azetidine-2-carboxylic acid (AZC) and incubated at 30 °C for six days. (**F**) As in (**E**), except cells were incubated on control media or media containing 0.05 µg/mL cycloheximide for two days.

A core Not4 activity is to regulate the assembly and activity of the proteasome to maintain ubiquitin homeostasis and control global proteostasis^20^. To determine the individual contributions the Not4 RING and RRM-C domains have in proteasome regulation, we prepared denaturing protein extracts from cells expressing the individual Not4 mutants, resolved them by SDS-PAGE, and performed α-ubiquitin IB. Very little ubiquitin specific smearing (indicative of polyubiquitylated protein accumulation) was detectable in WT control cells, while *not4Δ* accumulated significant amounts of polyubiquitylated proteins that was restored to normal by re-expressing Not4WT (Fig. 2A). Although the extent of accumulation exhibited minor variability between replicates, the Not4RING mutant consistently resulted in lower amounts of polyubiquitylated proteins relative to *not4Δ* vector control (Fig. 2A). These data demonstrate that the ligase deficient Not4RING mutant still promotes low level 19S RP-dependent deubiquitylation. Importantly, while the Not4RRM mutant alone had no effect, the Not4RING/RRM mutant completely phenocopied Not4 loss of function (Fig. 2A). Importantly, the proteasome associates both with Not4WT and the different mutants, thus excluding the possibility these changes in 19S activity are caused by impaired interactions between the 19S and Ccr4-Not (Supplementary Fig. S1). Thus, the Not4 RRM-C acts redundantly with the RING domain to control global 19S RP-dependent deubiquitylation.

We next assessed the catalytic activity of the 20S CP from cells expressing Not4WT and the different mutants by incubating equal amounts of cell extract with the fluorophore 7-amino-4-methylcoumarin (AMC) labelled substrate, LLVY-AMC. When cleaved by the proteasome, this reagent releases a quantifiable fluorescence signal^20^. As a control, WT cell extracts were mock treated or treated with the proteasome-specific inhibitor MG-132 which substantially inhibits the 20S CP (Supplementary Fig. S2), thus demonstrating this assay specifically monitors 20S CP activity. Consistent with previous observations^20^, the 20S CP activity from *not4Δ* control vector cells was increased relative to that from WT control, while 20S CP activity was restored to normal in *not4Δ* by re-expressing Not4WT (Fig. 2B). Although the Not4RING has impaired proteasome-dependent deubiquitylation (Fig. 2A), 20S CP activity in either the Not4RING or Not4RRM single mutants was similar to Not4WT. However, Not4RING/RRM expressing cells had 20S CP activity nearly identical to the *not4Δ* vector control (Fig. 2B). The differences in 20S CP activity were not due to altered expression of proteasome subunits since the 19S subunit Rpt2, and multiple 20S subunits, were expressed similarly between all samples (Fig. 2C). Therefore, Not4 regulates 20S catalytic activity redundantly through its RING and RRM-C domains.

Not4 positively regulates the epigenetic modification H3K4me3 by directly ubiquitylating the sole H3K4 demethylase Jhd2 to target it for proteasomal-dependent degradation^25,26^. We monitored H3K4me3 levels as an indicator of Not4-dependent Jhd2 ubiquitylation and turnover to assess the role the Not4 mutants had on *in vivo* substrate degradation. Not4 loss reduced H3K4me3, while re-expression of Not4WT, Not4RING, or Not4RRM, but not the Not4RING/RRM mutant, completely restored H3K4me3 (Fig. 2D). These data further demonstrate functional redundancy between the Not4 RING and RRM-C domains in regulating Not4-dependent substrate turnover. To further address the role these Not4 domains have in maintaining proteostasis, equivalent numbers of cells were spotted to control plates or plates containing 0.5 mg/mL azetidine-2-carboxylic acid (AZC) which induces protein misfolding and proteostatic stress^32^. While *not4Δ* is completely growth impaired, Not4WT restores growth (Fig. 2E). Intriguingly, the Not4RING exhibited a partial growth defect while both the Not4RRM and Not4RING/RRM were completely growth impaired (Fig. 2E). Furthermore, the Not4RRM caused a similar growth defect on low concentrations of cycloheximide which is a phenotype thought to indicate defects in ubiquitin homeostasis (Fig. 2F)^20,40^. These data thus demonstrate that the Not4 RING and RRM-C domains together function to regulate the 19S RP-dependent deubiquitylation and 20S CP catalytic activity required for sustaining global proteostasis.

### The Not4 RRM-C domain selectively regulates Pol II-dependent gene expression

**Figure 3:**
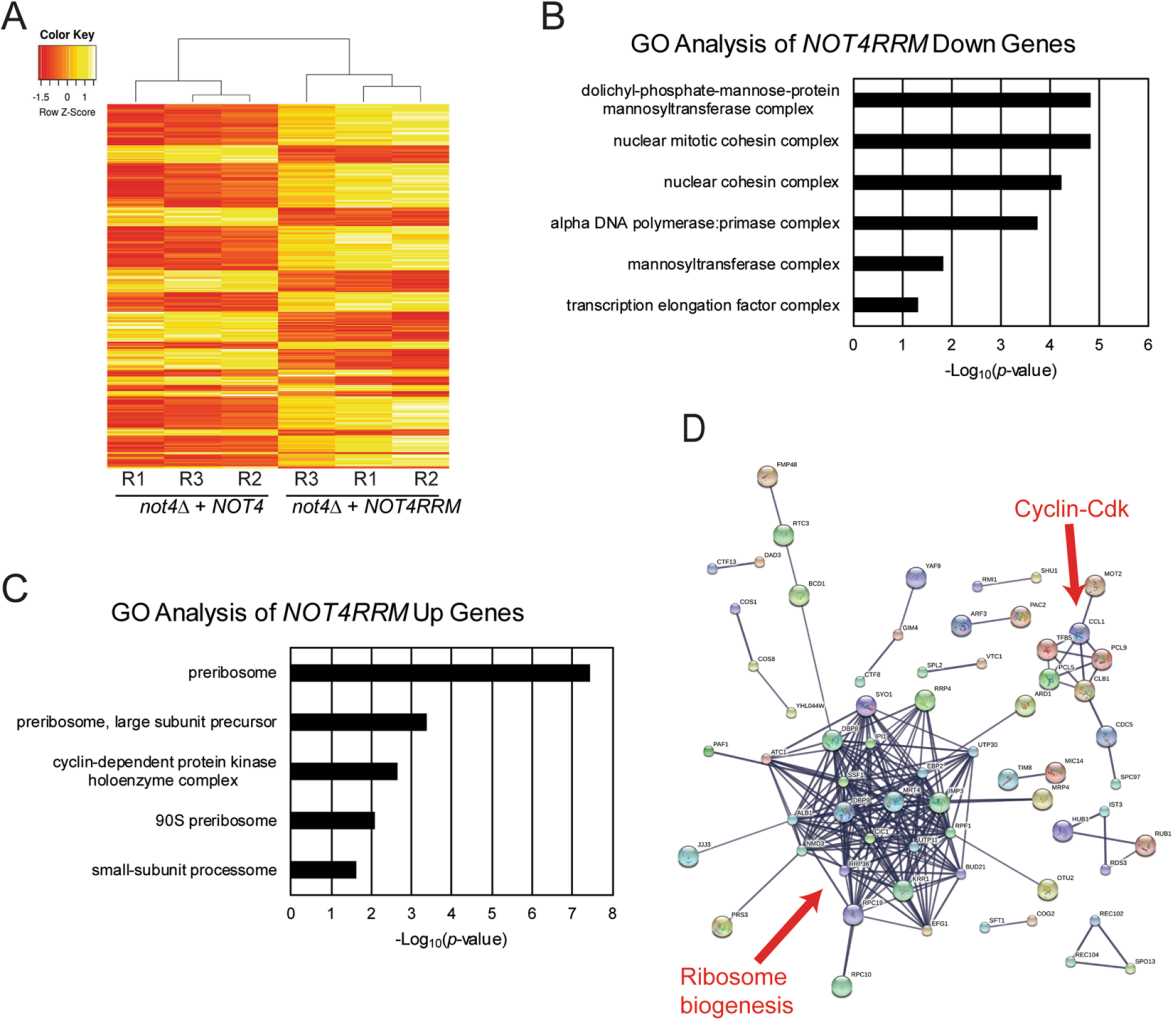
The Not4 RRM-C selectively regulates global gene expression. (**A**) Heat map of the 353 differentially regulated genes between Not4WT and Not4RRM expressing cells. Cultures were grown in triplicate, total RNA was isolated and utilized for RNA-seq as described in the Methods. R-replicate. (**B** and **C**) Gene Ontology (GO) analysis of those GO categories containing three or more genes identified in the downregulated (**B**) or upregulated (**C**) Not4RRM gene sets. Chi-squared analysis was used to determine the statistical significance for each GO category relative to their occurrence within the genome. (**D**) STRING analysis of the Not4RRM upregulated gene set. All 128 genes were submitted to the STRING database (https://string-db.org/) for high-confidence network analysis with the non-connected nodes removed.

To ascertain if the Not4 RRM-C also functions in Pol II-dependent gene regulation, *not4Δ* cells expressing Not4WT or the Not4RRM mutant were cultured in triplicate, total RNA was isolated, and RNA-seq was performed. Approximately 6% of all genes (353 total with a minimum of 1.5-fold or greater change in expression and a FDR of *p* < *0.05*) were differentially expressed in Not4WT and Not4RRM cells, with significant concordance between individual replicates (Pearson correlation coefficient, r = 0.96 or greater) (Fig. 3A and Supplementary Fig. S3). Gene ontology (GO) analysis revealed that the Not4RRM mutant selectively affected the expression of genes involved in specific biological processes, with the Not4RRM mutant causing a reduction in expression of 225 genes in GO categories for intracellular ribonucleoprotein complex, DNA polymerase/primase, mannosyltransferase activity, cohesin complex, and transcription elongation factor complex (Fig. 3B and Table 1). Intriguingly, within the transcription elongation category, *CCR4* expression was reduced in Not4RRM expressing cells, suggesting wild-type Not4 is required for optimal expression of its companion Ccr4-Not subunit through the RRM-C domain. Additional genes within this category include the transcription elongation regulators *SPT16* and *SPT5*, both of which encode Pol I and Pol II transcription elongation regulators^1^. These results suggest the Not4 RRM-C may promote Pol I and Pol II transcription elongation by controlling expression of these essential elongation factors.

**Table 1:** GO Analysis of *NOT4RRM* Differentially Regulated Genes.

GO ID	GO Category	Genes from RNA sequencing	*NOT4RRM* Gene Expression Change (Down Genes, 225 total)	P-value*
30529	Intracellular ribonucleoprotein complex	*CCR4, PAT1, SUB2, GCN2, EFT2, DUG1, XRN1, GCN1, SCP160, YNL247W, EFT1, ALE1, NEW1*	Down	0.004961
5840	Alpha DNA polymerase:primase complex	*SPT16, PRI2, POL1*	Down	0.0001755
785	Mannosyltransferase complex	*PMT5, PMT4, PMT3*	Down	0.01459
5816	Nuclear cohesin complex	*IRR1, SMC3, PDS5*	Down	0.00005885
790	Transcription elongation factor complex	*CCR4, SPT16, SPT5*	Down	0.04944
922	Nuclear mitotic cohesin complex	*IRR1, SMC3, PDS5*	Down	0.00001509
5819	Dolichyl-phosphate-mannose-protein mannosyltransferase complex	*PMT5, PMT4, PMT3*	Down	0.00001509
**GO ID**	**GO Category**	**Genes from RNA sequencing**	***NOT4RRM*** **Gene Expression Change (Up Genes, 128 total)**	**P-value**
30684	Preribosome	*KRR1, EFG1, CIC1, SSF1, IPI1, RPF1, IMP3, DBP8, NMD3, MRT4, UTP11, EBP2, UTP30, BUD21, RRP36*	Up	3.666E-08
30687	Preribosome, large subunit precursor	*CIC1, SSF1, IPI1, RPF1, NMD3, MRT4, EBP2*	Up	0.0004203
30686	90S preribosome	*KRR1, IMP3, DBP8, UTP30, BUD21, RRP36*	Up	0.00788
32040	Small-subunit processome	*KRR1, IMP3, UTP11, BUD21*	Up	0.02351
307	Cyclin-dependent protein kinase holoenzyme complex	*PCL9, CLB1, PCL5*	Up	0.002168

^*^Statistical significance determined by Chi-squared test.

Similar analysis of the upregulated genes (128 total) in Not4RRM expressing cells revealed GO categories for preribosome, large subunit preribosome precursor, 90S preribosome, small subunit processome, and cyclin-dependent protein kinase (CDK) activity to be significantly overrepresented in Not4RRM cells (Fig. 3C and Table 1). The genes in the CDK category encode two distinct cyclins (*PCL9* and *PCL5*) that bind the Cdk Pho85 to regulate cell-cycle and environmental nutrient responses, while the third cyclin (*CLB1*) encodes an M-phase cyclin that promotes mitotic progression^41,42^. Although ribosomal protein encoding genes are highly expressed in growing cells, the Not4RRM upregulated genes in the ribosome-related GO categories only included genes whose products regulate ribosomal assembly but are not ribosomal constituents (Table 1)^43^. To determine if these differentially expressed genes exhibit functional connectivity as part of a larger protein-protein interaction network, we subjected both the upregulated and downregulated gene sets to high confidence STRING analysis^44^. The Not4RRM upregulated genes involved in ribosome biogenesis exhibit a high degree of interconnectivity, while a less dense interaction network was identified for the genes connected to cyclin-Cdk function (Fig. 3D). Intriguingly, a similar analysis of the 225 downregulated gene set did not exhibit the same dense interconnectivity (Supplementary Fig. S4). Therefore, these data highlight a specific role for the Not4 RRM-C domain in regulating the optimal expression of genes in biological networks controlling transcription elongation and chromosomal cohesion, as well as CDK-dependent nutrient signaling and ribosome formation.

**Figure 4:**
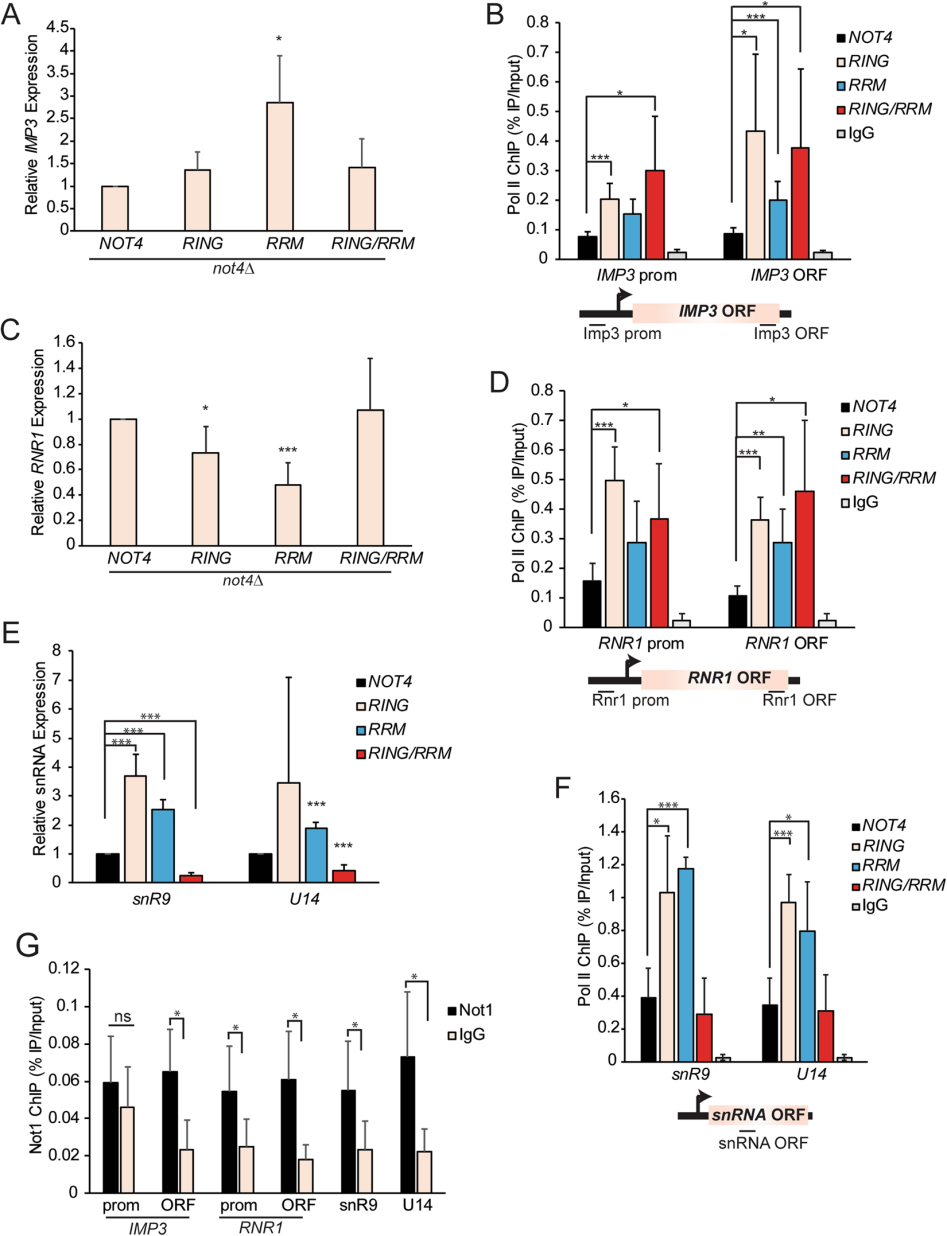
RNA polymerase II binding to Not4 regulated genes is repressed by the Not4 RING and RRM-C domains. (**A** and **B**) Gene expression (**A**) and Pol II ChIP (**B**) analysis of the Not4RRM upregulated gene *IMP3*. (**C** and **B**) Gene expression (**C**) and Pol II ChIP (**D**) analysis of the Not4RRM downregulated gene *RNR1*. (**E** and **F**) Expression (**E**) and Pol II ChIP (**F**) analysis of Not4 regulated snoRNAs. (**G**) ChIP for Not1 at the genes analyzed in (**A**–**F**). Data in (**A**,**C**,**E** and **F**) are the average and SD of five independent experiments. Data in **B**, **D**, and **G** are the average and SD of five independent experiments, while the data in (**F**) are the average and SD of four independent experiments. Statistical significance was determined by pairwise (relative to the Not4WT sample) two-sided Student’s t-test. *p < 0.05; **p < 0.01; ***p < *0.005*.

To validate the RNA-seq data, we examined by RT-qPCR the expression of a gene which increases (YHR148W, the *IMP3* gene) and one that decreases (YER070W, the *RNR1* gene) due to Not4RRM expression (Supplementary File 1). Consistent with the RNA-seq results, *IMP3* gene expression is upregulated in Not4RRM cells approximately 3-fold but it is not affected in either the Not4RING or Not4RING/RRM expressing cells (Fig. 4A). ChIP for Pol II at *IMP3* did not reveal a statistically significant increase for Pol II at the *IMP3* promoter, but it did demonstrate higher Pol II levels at the 3′ end of the *IMP3* ORF (Fig. 4B). Interestingly, while the Not4RING and NOT4RING/RRM mutants had no impact on *IMP3* mRNA (Fig. 4A), both mutants increased Pol II binding at the promoter and 3′ end of the *IMP3* ORF (Fig. 4B). Analysis of *RNR1* expression demonstrated that Not4RRM reduced *RNR1* mRNA levels (Fig. 4C), thus supporting our RNA-seq results. Expression of the Not4RING mutant only marginally reduced *RNR1* expression relative to Not4WT (*p* = *0.045* by two-tailed Student’s t-test), whereas the Not4RING/RRM mutant restored *RNR1* expression to normal (Fig. 4C). However, Pol II binding at the *RNR1* locus increased at the promoter in both Not4RING and Not4RING/RRM expressing cells, while Pol II was increased specifically at the *RNR1* 3′ end in Not4RRM cells (Fig. 4D). These data suggest a complex role for the different Not4 domains in regulating Pol II transcription, and that changes in Not4 regulated mRNA expression do not always correlate with the amount of Pol II bound. This disconnect is likely due to compensatory changes to mRNA levels at later stages of the mRNA lifecycle since Ccr4-Not is implicated in mRNA imprinting^45,46^.

Not4 loss upregulates several Pol II transcribed non-coding RNAs, including many small nucleolar RNAs (snoRNAs)^47^. Since these RNAs were not included in our RNA-seq experiment, we analyzed the expression of the representative snoRNAs *snR9* and *U14* to determine the impact the Not4 mutants had on their expression. Interestingly, we found that both *snR9* and *U14* were upregulated in the individual Not4RING and Not4RRM mutants, but their expression was significantly reduced in the Not4RING/RRM double mutant (Fig. 4E). Ccr4-Not control of these snoRNAs originally was suggested to involve post-transcriptional mechanisms involving interactions with the TRAMP and exosome complexes^47^, yet whether Ccr4-Not also regulated Pol II binding at these genes was not determined. We addressed this by analyzing Pol II at the ORFs for both genes and found that Pol II levels were significantly higher in Not4RING and Not4RRM compared to Not4WT, while the Not4RING/RRM mutant restored Pol II binding to that detected in Not4WT expressing cells (Fig. 4F). These data suggest that the Not4 RING and RRM domains may act individually as negative regulators of Pol II-dependent snoRNA transcription, yet disruption of both domains likely impairs additional Ccr4-Not functions necessary for sustaining snoRNA expression. The role of Not4 in regulating expression of all the genes analyzed is likely direct since we detected Ccr4-Not bound to either the promoters and/or gene bodies (Fig. 4G). Therefore, the Not4 RING and RRM-C domains have opposing functions on the steady-state expression of some mRNA genes (*IMP3* and *RNR1*), while they appear to individually repress, but collectively sustain, steady-state snoRNA expression. Since the impact these domains have on mRNA or snoRNA expression does not always correlate with Pol II binding, they likely contribute to Pol II-dependent gene expression at both the transcriptional and post-transcriptional levels.

**Figure 5:**
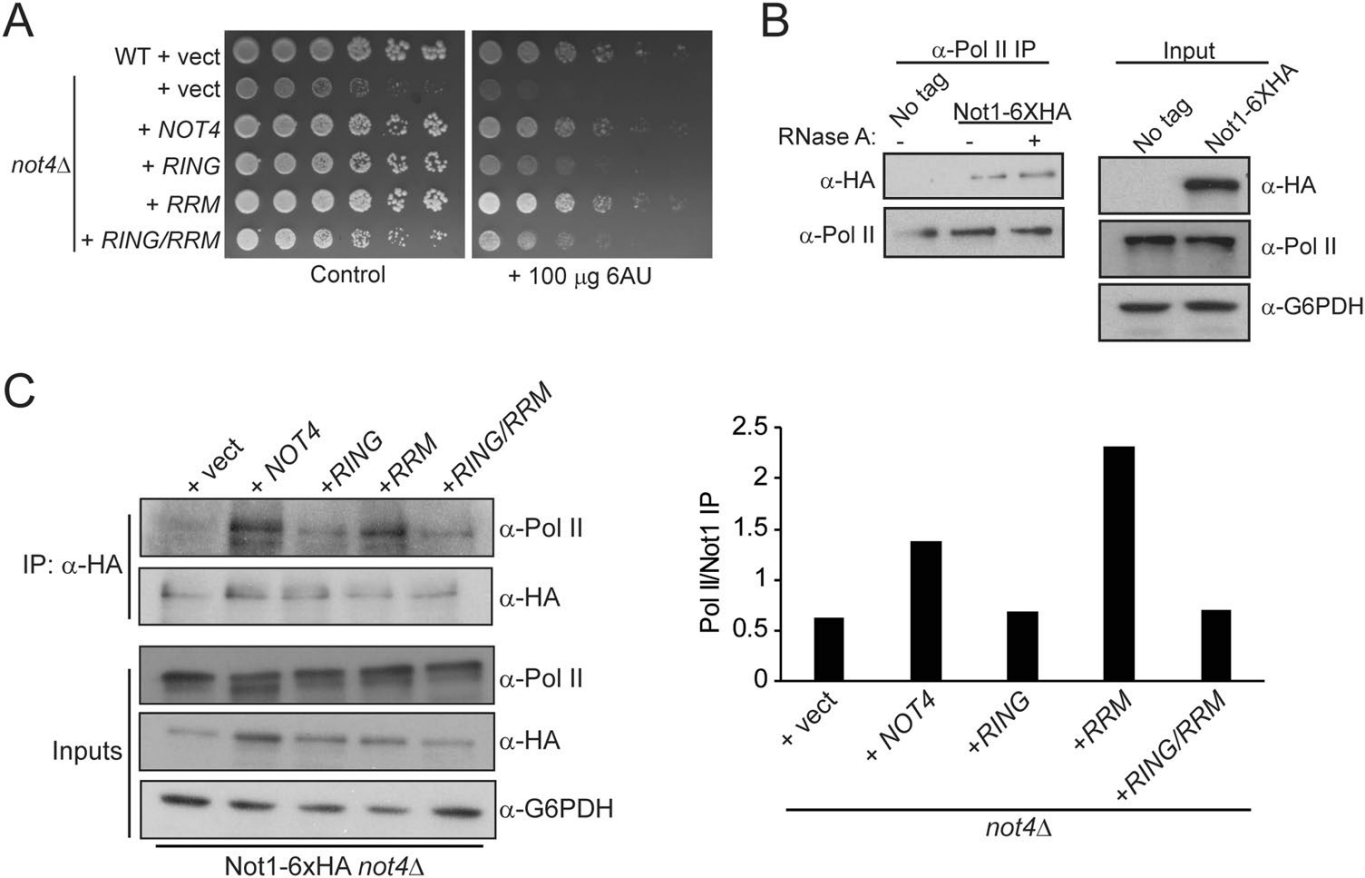
The Not4 RING domain regulates Ccr4-Not association with RNA Pol II. (**A**) The indicated strains were cultured overnight before 5-fold serially diluting equal numbers of cells and spotting to the indicated plates. Data were acquired after three days at 30 °C. (**B**) Cell extracts were prepared from a no tag control or Not1-6XHA expressing cell. RNA Pol II was immunoprecipated, washed once with IP buffer, and then incubated with 700 U/mL RNase A for 30′. Samples were washed twice more with IP buffer, and then subjected to 8% SDS-PAGE and immunoblot analysis with the indicated antibodies. (**C** and **D**) Extracts were prepared from a Not1-6XHA *not4Δ* strain expressing control vector or the indicated Not4WT or Not4 mutants, and then used for α-HA immunoprecipitation. The presence of co-precipitated RNA Pol II was detected by immunoblot and quantified as the ratio of Pol II signal to total Not1 precipitated. The data are representative of a minimum of three independent experiments.

To further address the role of the Not4 RRM-C domain in transcriptional regulation, we analyzed sensitivity of *not4Δ* cells expressing Not4WT, Not4RING, Not4RRM, or Not4RING/RRM to the transcription elongation inhibitor 6-azauracil (6AU). Consistent with previous reports, *not4Δ* is 6AU sensitive^48^, while Not4WT expression restores growth to normal (Fig. 5A). Intriguingly, only the Not4RING and the Not4RING/RRM resulted in 6AU sensitivity, albeit not to the same extent as a *not4Δ*. These data demonstrate that the critical role for Not4 in transcription elongation requires the RING, but not the RRM-C, domain (Fig. 5A). Ccr4-Not interacts with the elongating Pol II complex through the Rpb4/7 submodule, an interaction originally defined as independent of RNA binding^31,49^. Due to the transcriptional effects caused by the Not4RRM mutation, we re-evaluated whether RNA might affect Ccr4-Not association with Pol II by assaying for the presence of Ccr4-Not from mock treated or RNase A-treated Pol II immunoprecipitates. Consistent with these previous reports^31,49^, we found Ccr4-Not association with Pol II is RNA independent (Fig. 5B). As such, the Not4RRM effect on Pol II transcription cannot be explained by impaired Not4 binding to RNA.

To determine whether the Not4 RRM-C or RING domains contribute to Ccr4-Not association with Pol II, we immunoprecipitated Not1 from *not4Δ* cells with control vector or vector expressing Not4WT, Not4RING, Not4RRM, or Not4RING/RRM. The immunoprecipitates then were probed for the presence of Pol II. While only background levels of Pol II associated with Ccr4-Not in the absence of Not4, Not4WT expression restored robust Pol II association (Fig. 5C). Although Not4RRM expression had no effect on Ccr4-Not association with Pol II, both the Not4RING and Not4RING/RRM dramatically reduced these interactions (Fig. 5C). Collectively, these data demonstrate that while the Not4 RRM-C domain has highly specific effects on Pol II-dependent gene expression, the RING domain plays a key role in promoting Ccr4-Not interactions with the Pol II holoenzyme.

## Discussion

In this report, we provide multiple lines of evidence that the uncharacterized Not4 RRM-C domain acts in conjunction with the RING domain to mediate Not4 *in vivo* activities necessary for proteasome function and Pol II transcription. We also demonstrate that the Not4 RRM-C domain controls a highly selective Pol II-dependent gene expression program, thus demonstrating its role in regulating Pol II gene expression is very specific. We further demonstrate that the RING and RRM domains act individually as general negative regulators of Pol II binding to target genes; however, the increased Pol II binding in RING and RRM-C mutants does not always correlate with changes to the corresponding mRNA. These results suggest Not4 likely controls their post-transcriptional regulation as well, a phenomenon consistent with the role of Ccr4-Not in controlling all aspects of the mRNA lifecycle^2,3^. Although the RNA recognition motif is a well-defined RNA binding motif, some RRMs do not bind RNA but instead serve as protein-protein interaction motifs^50^. Determining if the Not4 RRM-C binds to RNA *in vivo* or if it mediates protein-protein interactions remains an area of ongoing study.

Not4, via its RING domain, controls proteasome assembly and activity in part by maintaining the stability of the proteasome associated factor Ecm29^20^. However, this mechanism does not explain all Not4-dependent proteasome control since we detect less severe accumulation of polyubiquitylated proteins in a Not4RING mutant relative to a *not4Δ*. These observations are therefore consistent with the original study that determined the role of Not4 in proteasome regulation extends beyond control of Ecm29 stability^20^. Our data demonstrate that both the Not4 RRM-C and RING domains function redundantly in 19S and 20S regulation since only mutation of both simultaneously reproduces the severity of proteasomal defects found in *not4Δ*. These results suggest that this previously uncharacterized Not4 domain has an important and unappreciated role in regulating proteostasis, perhaps involving these uncharacterized, Ecm29-independent mechanisms. As previously demonstrated, Not4 must integrate into Ccr4-Not to regulate the proteasome^20^. An intriguing speculation is that the activity of other Ccr4-Not subunits, specifically Ccr4-dependent mRNA processing as this subunit is spatially proximal to Not4 within Ccr4-Not^8^, may direct Not4 via the RRM-C to RNA substrates that link mRNA turnover to proteasome regulation. If so, proteasome assembly and global proteostasis control may be intertwined with global mRNA decay via Ccr4-Not.

Our data further demonstrate that the Not4 RRM-C regulates only a small (~6%), non-random subset of the cellular transcriptome. GO analyses of the affected transcripts demonstrate that the Not4 RRM-C positively controls mRNA expression of specific transcription elongation factors, including *CCR4*. Although the implications of this regulation are not clear, one possibility could be that defects in Not4 activate a negative feedback mechanism that represses expression of additional Ccr4-Not subunits. Given its especially pronounced role in global transcription elongation and mRNA decay, such a mechanism could minimize the impact dysfunctional Ccr4-Not complexes have on the cellular transcriptome. Not4 RRM-C disruption increases expression of genes functioning in biological networks regulating nutrient responses, including those involved in cell-cycle control and nutrient signaling by the Pho85 CDK pathway, as well as ribosomal biogenesis genes. Consequently, it is likely *in vivo* that manipulating Not4 activity will be a key aspect of the cellular response to nutrient availability required for increasing cellular biosynthetic capacity. Such a role agrees with our previous study demonstrating that Ccr4-Not functions as an RNA Pol I transcriptional co-regulator necessary for ribosomal RNA biogenesis^9^.

Although both the Not4 RING and RRM-C contribute to proteasome regulation, how they affect Pol II-dependent gene regulation is less clear. Our gene-specific analyses validated the RNA-seq data in that the RRM-C domain negatively (*IMP3*) or positively (*RNR1*) regulated the steady-state expression of these genes. Intriguingly, on these same genes we find that the RING domain had minimal or no effect on their expression, yet when combined with the RRM-C mutant the double mutant opposed the changes in gene expression caused by RRM-C disruption alone. While the RING mutant did not alter their expression, it increased Pol II levels at the promoter and 3′ end of these genes suggesting wild-type Not4 negatively regulates Pol II binding. This interpretation would be in line with the role Ccr4-Not has as a negative transcriptional regulator, and it would be consistent with our results demonstrating Ccr4-Not requires the Not4 RING domain to associate with Pol II. The RRM-C mutant had a lesser effect on Pol II binding, with Pol II increasing only at the 3′ end of these genes. These results suggest the possibility that Not4RRM cells may have a mild transcription elongation and/or termination defect. Clearly, in the individual and combinatorial mutants we detect a discordance between the levels of Pol II bound to these genes and their steady-state expression. Although the mechanism explaining these differences is under investigation, we believe the most likely explanation is that Ccr4-Not also functions at a post-transcriptional stage to regulate the stability of these RNAs which would be consistent with its known functions^2,3^.

A previous report demonstrated that Not4 loss increased steady-state expression of snoRNAs, a phenomenon that was attributed to their decreased turnover^47^. We find that snoRNA expression increases upon either RING or RRM-C disruption, but a combinatorial mutant dramatically impairs snoRNA expression. The differences in our results compared to those previously published is that we have generated a system that impairs Not4 function without complete subunit loss. In the previous study, a *not4Δ* likely affected the stability of additional Ccr4-Not subunits which is an observation that was recently reported^51^. As such, while a *not4Δ* may increase snoRNA expression by impairing additional Ccr4-Not regulated activities, our use of Not4 point mutants presumably minimizes disruption of these other functions.

Collectively, we provide evidence that the unique Not4 ligase, which is the only eukaryotic RING ligase containing both an RRM and a C3H1 domain, utilizes these domains to regulate its *in vivo* activities. Going forward it will be key to define if the RRM-C binds RNA, and if it does so, determine how RNA binding impacts Not4-dependent substrate ubiquitylation. Alternatively, if these domains do not function in RNA binding, could they play a role in substrate recognition by the ligase? Defining novel Not4 substrates, and how the RRM-C recognizes them within Ccr4-Not, will be essential for understanding how Ccr4-Not contributes to such a diversity of biological processes.

## Materials and Methods

### Yeast strains and growth conditions

All strains used in this study are in the BY4741 genetic background. Genetic manipulations to generate genomically-tagged derivatives were performed using standard approaches^52^. Cells were cultured in synthetic complete (SC) media (0.17% YNB without amino acids or ammonium sulfate, 0.1% glutamic acid, 2% glucose, 0.2% amino acid dropout mix) with all key nutrients except for uracil (to select for plasmid maintenance) added back. Unless stated otherwise, cultures were grown to an OD_600_ = 1.0–1.6 before harvesting. All yeast media reagents were purchased from US Biologicals and Research Products Incorporated. Yeast strains used in this study are listed in Supplementary Table S1.

### Cloning

The wild-type *NOT4* open reading frame was amplified as a C-terminal mono-FLAG fusion using Q5 enzyme (New England Biolabs) and cloned into the Xba1 and Eco RI digested plasmid p416*ADH*^53^. Mutations were introduced using the QuikChange Mutagenesis kit from Agilent Technologies and confirmed by sequencing. Plasmids are listed in Supplementary Table S1.

### Chromatin immunoprecipitation (ChIP), quantitative PCR (qPCR), and RNA isolation

ChIP was performed by growing the indicated strains in 200 mL SC-uracil media and cross-linked with formaldehyde at a final concentration of 1% for 15 minutes followed by a quench with glycine at 125 mM final concentration for five minutes. Cell pellets were lysed in in 300 mM FA lysis buffer (50 mM HEPES-KOH pH 7.5, 300 mM NaCl, 1 mM EDTA, 1% Triton X-100, 0.1% sodium deoxycholate) by bead beating. Samples were then sonicated using a Qsonica probe sonicator with a 1/8″ probe using the following settings: 4′ at 50% amplitude and 30″ on/30″ off cycle. Sonicated samples were clarified by centrifugation at 4,000xg for 15′ at 4 °C and then the soluble chromatin fraction was quantified by Bradford assay. Typically, 300–500 µg of chromatin extract was incubated with the appropriate antibody overnight at 4 °C, with 10% of the total extract set aside as an input control. Immune complexes were captured by incubation with Protein A agarose beads, washed extensively as described previously^9^, and DNA then eluted. Samples were incubated for four hours to overnight at 65 °C in the presence of high salt to reverse cross-links, and then the DNA was purified. ChIP IPs were eluted in 50 µL, and inputs in 100 µL, molecular grade water, and they were analyzed by qPCR using an Applied Biosystems StepOne Plus Real-time PCR machine. The following formula was used to calculate IP/Input percent enrichment: (=2^(Input Ct-IP Ct)^×100).

Gene expression analysis was performed using total RNA isolated from cells by bead beating in TriReagent (Sigma). The total RNA was digested with DNase I (Promega), phenol/chloroform extracted, and then resuspended in RNase free water. To generate cDNA, 1 µg total RNA was used with random hexamer primers and the ImProm II cDNA synthesis kit (Promega) in a 20 µL reaction volume per the manufacturer’s protocol. After cDNA synthesis, samples were then raised to a final volume of 100 µL, and 1 µL was used for qPCR. Gene specific targets were normalized to the *SPT15* housekeeping gene using the formula(=2^(*SPT15* Ct- Gene target Ct)^), and the Not4RRM sample was expressed relative to the Not4WT sample which was set to a value of 1. All primers used for ChIP and gene expression analyses are listed in Table S2. Statistical analyses were performed using Microsoft Excel.

### RNA sequencing (RNA-seq)

Submitted total RNA samples were analyzed on an Agilent Bioanalyzer and spectrophotometric analysis to determine RNA quality. After passing this initial screening 500 ng of total RNA was used to prepare libraries for sequencing using the Lexogen SENSE mRNA-seq library kit for Ion Torrent. Libraries were amplified for 12 cycles as the final step of library preparation. Before sequencing, small aliquots of this material were quantified by qPCR utilizing the KAPA Library Quantification kit for the Ion Torrent. Quantification of data from the qPCR was used to balance the barcodes for final pooling before sequencing. Following this final pooling, the library pools were sized to a target size of 300 bp on a Pippin Prep instrument. The sized libraries were examined on an Agilent High Sensitivity DNA chip, quantified using real-time PCR, and used for sequencing on an Ion Torrent Proton.

### Bioinformatics Analysis of RNA-Seq data

The adapter-free FASTQ files were collected from the Ion Torrent Proton, and all FASTQ files were subjected to quality assurance using FASTQC (http://www.bioinformatics.babraham.ac.uk/projects/fastqc/). All reads were trimmed to remove low quality data (phred score < 20). After quality assurance trimming, all samples were aligned to the S288C *Saccharomyces* transcriptome using RNA-STAR, and aligned read counts were mined for each transcript (http://www.ncbi.nlm.nih.gov/pubmed/23104886). After gathering read counts, each sample was scaled to the lowest coverage in order to remove any bias from sequencing depth difference. This resulted in each sample having 14 million aligned read counts, which then were analyzed for differential gene expression between cells expressing control and mutant Not4. Mean, variance, standard deviation and standard error of means were then calculated. The fold change and *p*-value for each gene were calculated using a two-sided Student’s *t* test assuming unequal variance. A fold change greater than fifty percent and a *p*-value less than 0.05 was used for differential expression cutoffs. The resulting list was subjected to the Benjamini-Hochberg false discovery method^54^. Only genes with a FDR less than 0.05 were allowed into the final list, which then was loaded into DAVID for functional annotation^55,56^. The functional classification list was analyzed by chi-squared analysis in order to identify any categorical groups that were significantly different.

### Immunoprecipitation and Immunoblotting

For standard immunoprecipitation and immunoblotting analysis, log phase cell extracts were prepared in immunoprecipitation buffer (150 mM NaCl, 10% glycerol, 10 mM Tris, pH 8.0, and 0.1% NP-40 containing protease and phosphatase inhibitors and 1 mM DTT) by bead beating, clarified, and then quantified by Bradford assay. Approximately 500 µg of total extract was rotated at 4 °C for a minimum of one hour to overnight for immunoprecipitations. Immune complexes were captured using Protein A conjugated agarose beads and washed extensively before resolving by SDS-PAGE. Samples were transferred to PVDF membrane and immunoblotted with the indicated antibodies. For the ubiquitin analyses, log phase yeast cell pellets were resuspended in denaturating buffer (8 M urea, 300 mM NaCl, 50 mM Tris pH 8.0, 0.5% Triton X-100) and the cell suspension was disrupted with glass beads for 3 min at 4 °C. Samples were resolved by 7.5% SDS PAGE gel and analyzed by immunoblotting. Immunoblot quantifications were performed by ImageJ analysis. Uncropped images of all immunoblots can be found in Supplementary Figs 5–9.

### 20S Proteasome Activity Assay

The Chemicon Proteasome Activity Assay Kit was used per the manufacturer’s instructions. Briefly, log phase cell extracts were prepared as described above and 100 µg cell extract was incubated for 2 hours at 37 °C in activity assay cocktail. Fluorescence was detected utilizing a 96-well Molecular Device spectraMax M2e fluorescence plate reader with a 380/460 nm filter set.

### Antibodies

Antibodies used are as follows: α-HA (Santa Cruz, sc-7392), α-ubiquitin (Santa Cruz, sc-8017), α-FLAG (Sigma, #F1804), α-G6PDH (Sigma, #A9521), α-H3 (Active Motif, #39163), α-H3K4me3 (Active Motif, #39159), α-RNA Polymerase II (Active Motif, #39097), α-Rpt2 (Enzo Life Sciences, #BML-PW8260), and α-20S (Enzo Life Sciences, #BML-PW8195). The α-Rpt6 antibody was described previously^25^.

### Data availability

Sequencing data have been uploaded to the NCBI’s GEO database and are available using the accession number GSE102192. All other data generated in this study are included in the published article or are available in the associated supplementary material. All yeast strains and plasmids generated in this study are available to the scientific community upon request.

## Electronic supplementary material

Supplementary Information
