# Supplementary material for: The conserved RNA recognition motif and C3H1 domain of the Not4 ubiquitin ligase regulate in vivo ligase function
Source: Sci Rep. 2018 May 25;8:8163. doi: 10.1038/s41598-018-26576-1 (PMC5970261; doi:10.1038/s41598-018-26576-1)
Supplement: Supplementary file 1 — Supplementary Information [file 41598_2018_26576_MOESM1_ESM.pdf]

Supplemental Information for:

The conserved RNA recognition motif and C3H1 domain of the Not4 ubiquitin ligase regulate *in vivo* ligase function

Hongfeng Chen[#], Tirupataiah Sirupangi[#], Zhaohui Wu[#], Daniel L. Johnson[†], and R. Nicholas Laribee[#*]

[#] *Department of Pathology and Laboratory Medicine, and Center for Cancer Research, University of Tennessee Health Science Center;* [†]*Molecular Bioinformatics Core and the University of Tennessee Health Science Center Office of Research, University of Tennessee Health Science Center, Memphis, TN, 38163 United States of America.*

A

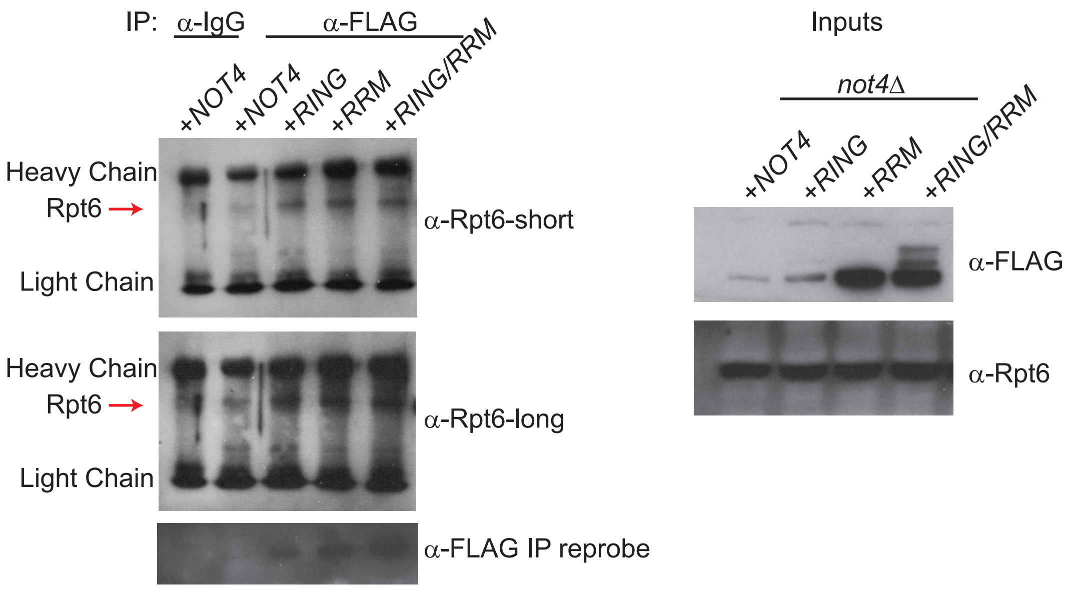

B

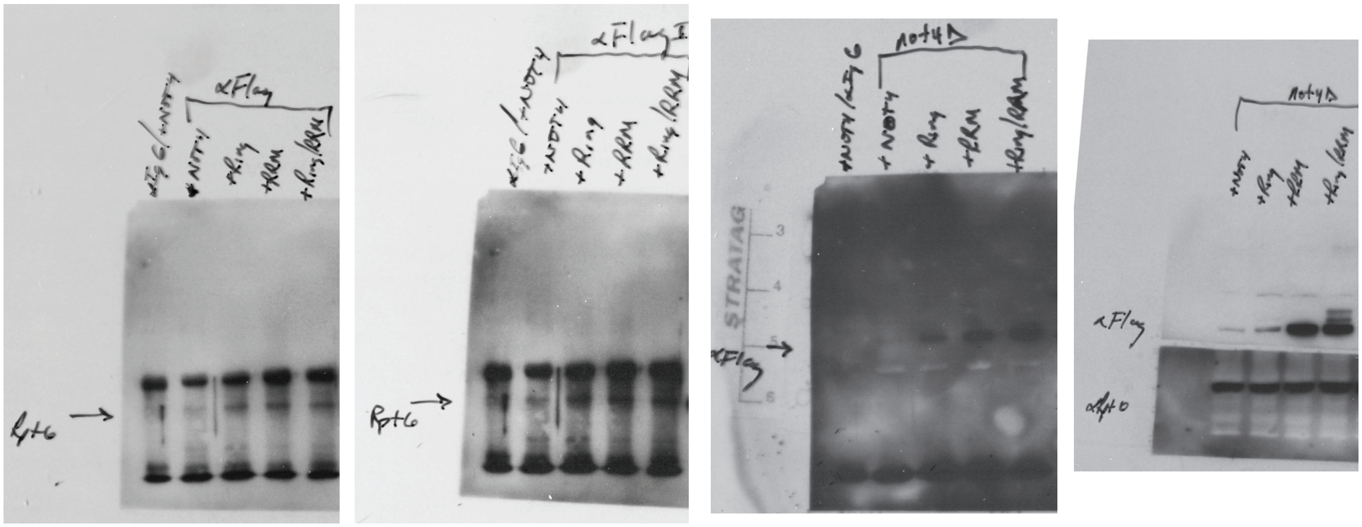

Supplementary Figure S1. Not4WT and the Not4 mutants associate with the proteasome. A. Not4 IP and immunoblot for the 19S proteasome subunit Rpt6. **B.** Raw immunoblots from **A.**

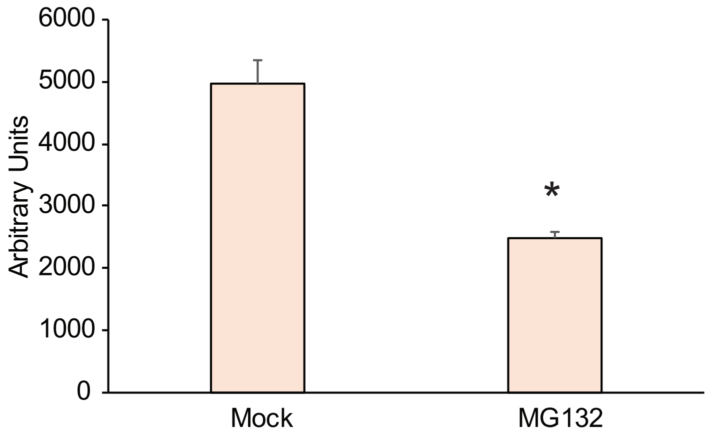

Supplementary Figure S2. Proteasome activity is sensitive to inhibition with the proteasome -specific inhibitor MG132. Extracts from wild-type cells were mock treated or treated with 50 μM MG132 for one hour before performing the activity assay as outlined in the Methods. Data are the average and SD of two independent experiments with significance determined by two-sided Student's t-test. *- $p < 0.05$.

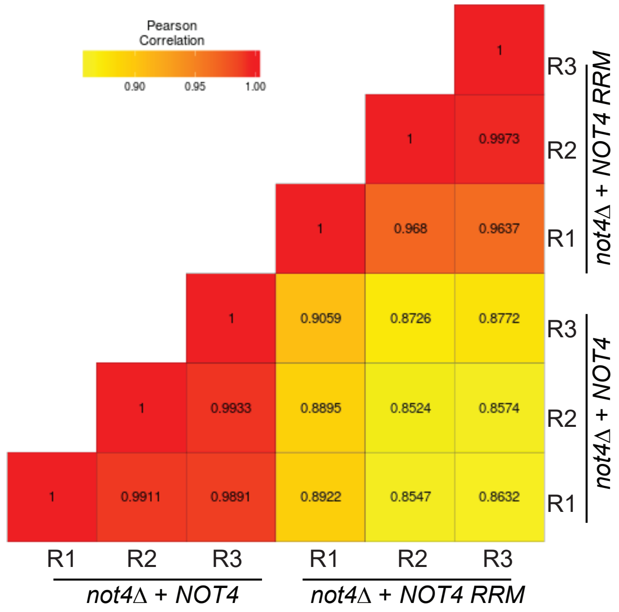

Supplementary Figure S3. Pearson correlation analysis of Not4RRM RNA-seq data. The RNA-sequencing results from the triplicate cultures of not4   cells expressing either Not4WT or Not4RRM were subjected to Pearson correlation analysis. R- replicate.

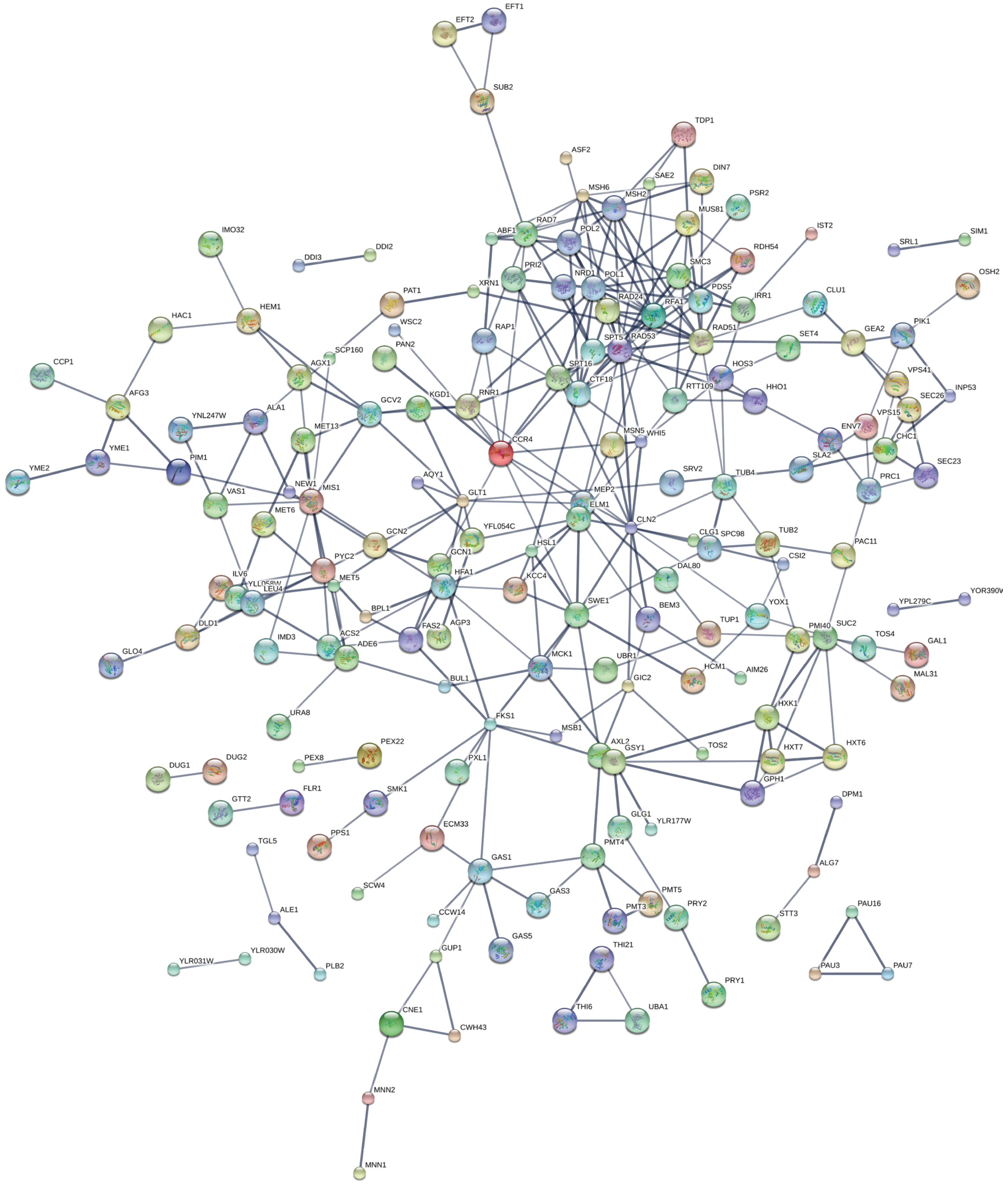

Supplementary Figure S4. STRING analysis of the Not4RRM downregulated gene set. STRING analysis of the Not4RRM downregulated gene set. All 225 genes were submitted to the STRING database (https://string-db.org/) for high-confidence network analysis with the non-connected nodes removed.

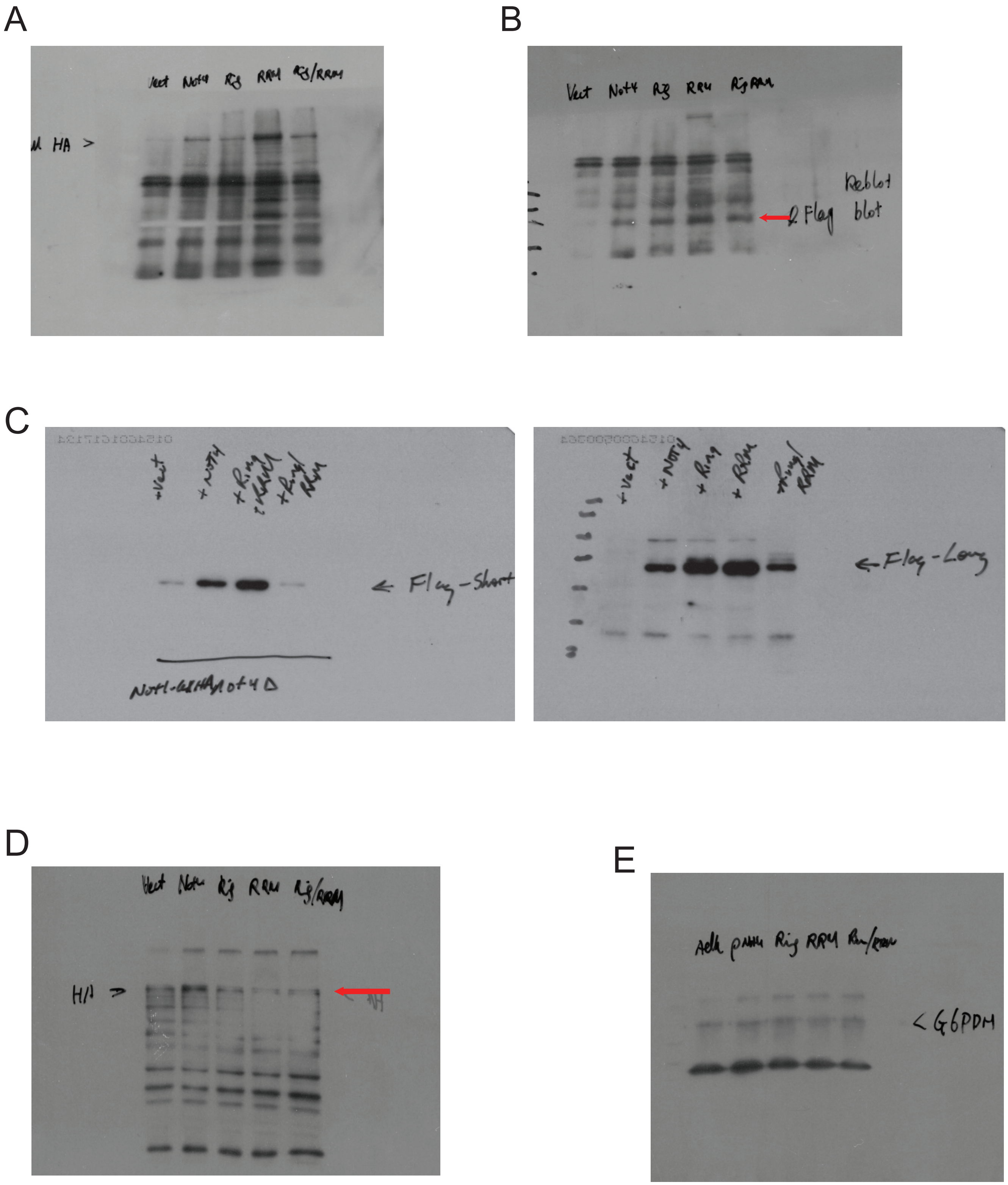

Supplementary Figure S5. Full-length immunoblots for Figure 1E. A and **B.** Blots from immunoprecipitation. **C-E.** Input blots. Note that the red arrows in panels **B** and **D** denote Not4-FLAG (**B**) and full-length Not1-6XHA (**D**).

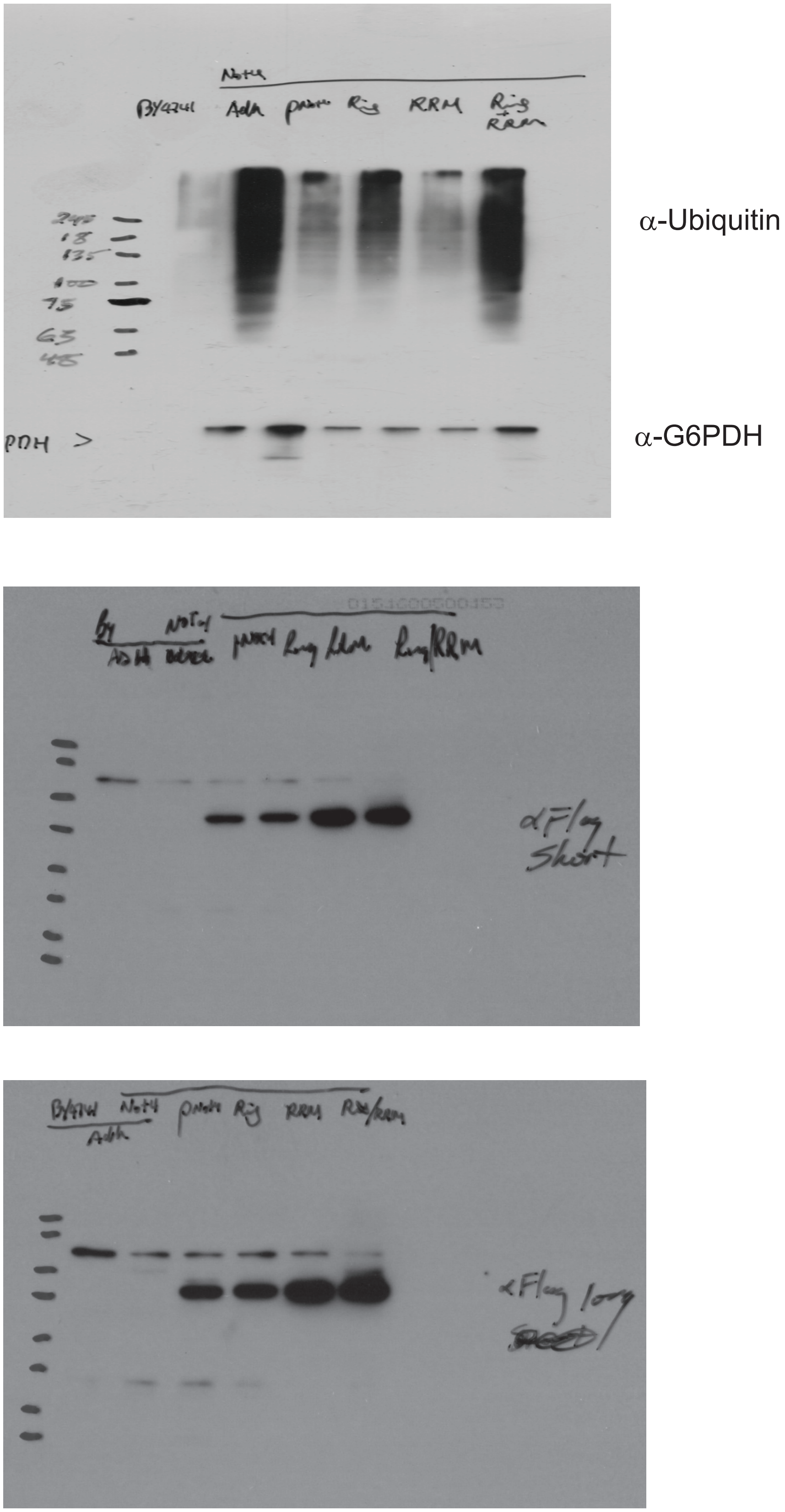

Supplementry Figure S6. Full-length immunoblots for Figure 2A.

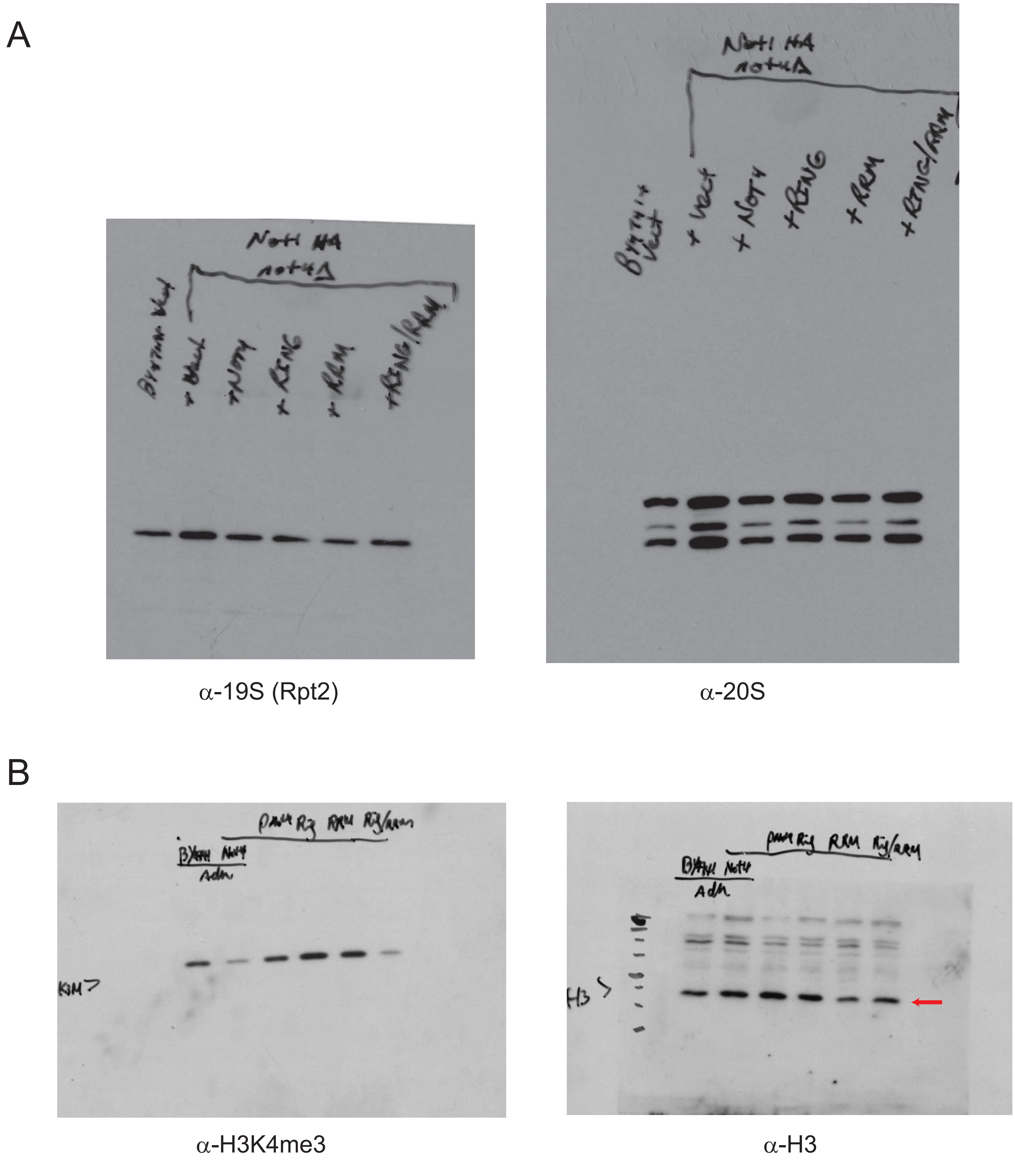

Supplementary Figure S7. Full-length immunoblots for Figure 2C and 2D. A. Immunoblots for proteasome subunits in Figure 2C. **B.** Immunoblots for histone from blots in Figure 2D. Red arrow denotes the histone H3 specific band.

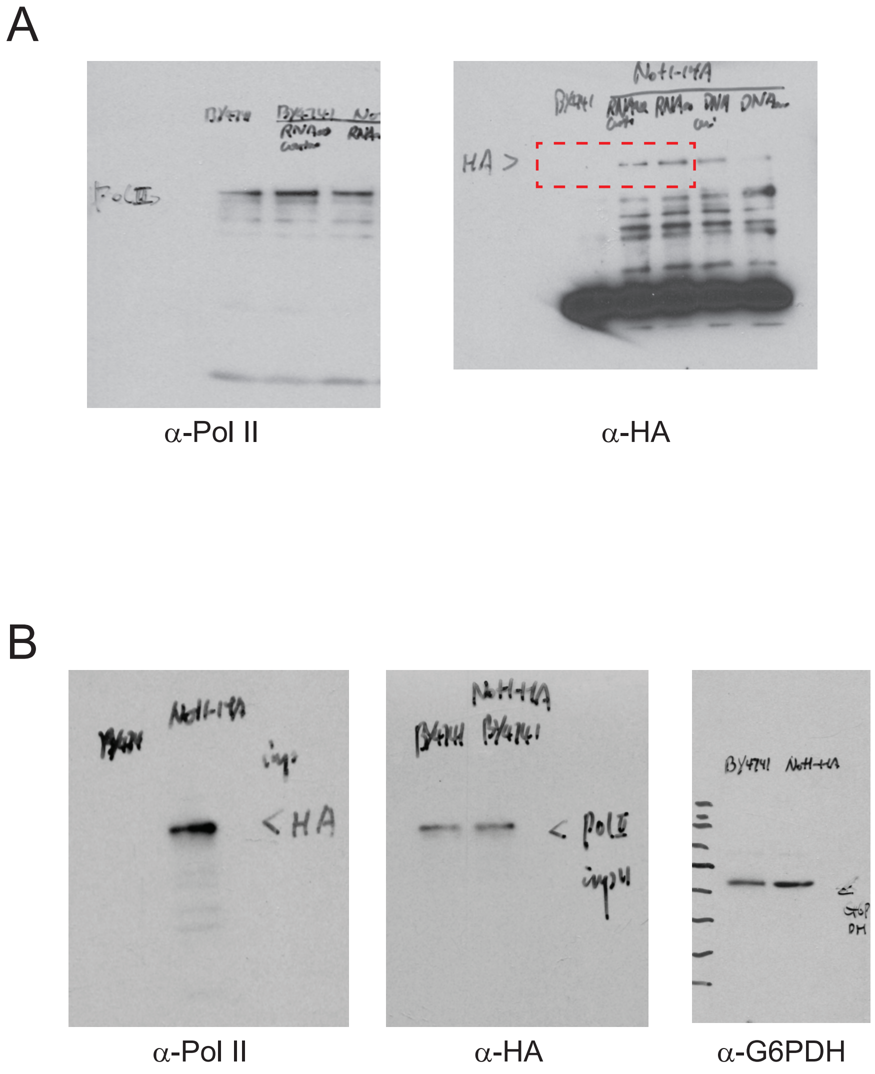

Supplementary Figure S8. Full-length immunoblots for Figure 5B. A. Immunoblots for the immunoprecipitation data in Figure 5B. Note that the boxed area indicates the α-HA presented in 5B. **B.** Full-length blots for the input results in Figure 5B.

A

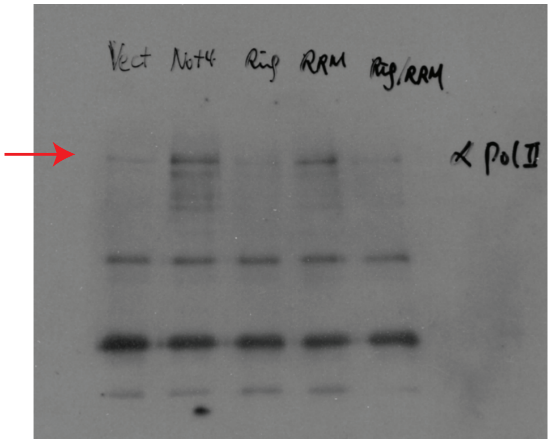

α-Pol II

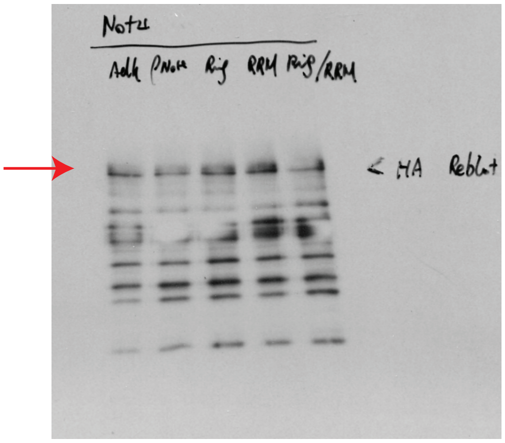

α-HA

B

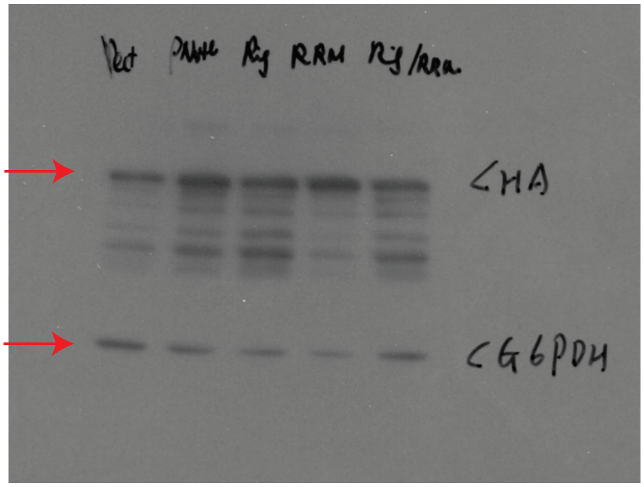

α-HA (top band)

α-G6PDH (bottom band)

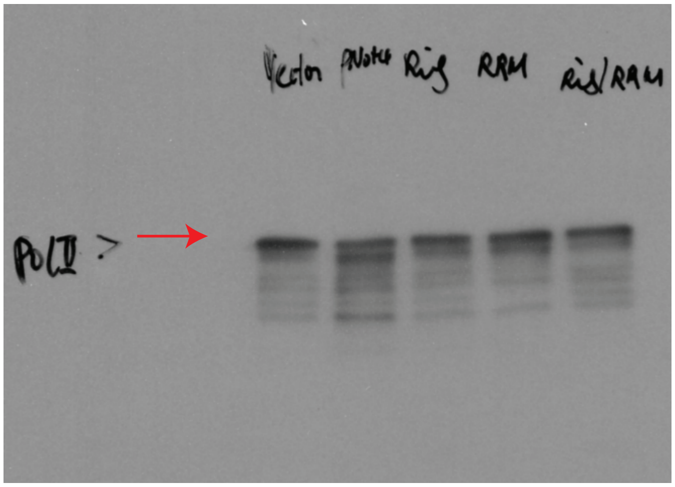

α-Pol II

Supplementary Figure S9. Full-length immunoblots for Figure 5C. A. Immunoblot data from the immunoprecipitation results shown in Figure 5C. **B.** Input blots for Figure 5C. Note for both A and B, red arrows denote the results presented in Figurer 5C.

Supplementary Table S1. Yeast strains and plasmids.

Strain	Genotype	Source
BY4741	MATa *his3Δ1 leu2Δ0 met15Δ0 ura3Δ0*	Open Biosystems
not4Δ	BY4741; *not4*Δ::KANMX	Open Biosystems
YNL794	BY4741; *NOT1-6XHA::HphNT1*	This study
YNL796	*NOT1-6XHA::HphNT1*; *not4*Δ::KANMX	This study
**Plasmid**	**Description**	**Reference**
p416ADH	*AmpR CEN6/ARSH4 URA3 ADH1prom; CYC1term*	(33)
pNOT4	*AmpR CEN6/ARSH4 URA3 ADH1prom-NOT4-FLAG; CYC1term*	This study
pNOT4RING	*AmpR CEN6/ARSH4 URA3 ADH1 prom-NOT4 I64A-FLAG; CYC1 term*	This study
pNOT4RRM	*AmpR CEN6/ARSH4 URA3 ADH1 prom-NOT4 G167A//F202A/C244A-FLAG; CYC1 term*	This study
pNOT4RING/ RRM	*AmpR CEN6/ARSH4 URA3 ADH1 prom-NOT4 I64A/G167A//F202A/C244A-FLAG; CYC1 term*	This study

Supplementary Table S2. Primers used in this study.

Primer	Sequence	Reference
NOT1 S2	TATATTTTTTTTTCTGAAATCATGATTTTCGTATATAAATAAATGCAGTTTTTATCGATGAATTCGAGCTCG	This study
NOT1 S3	GATGACCAATCCGCCACCATCAATAGAAGGCAAACCCCTCTACAATCCAACGCACGTACGCTGCAGGTCGAC	This study
qNOT4 5'for	AAGCAATCCACAACGCCTTA	This study
qNOT4 5'rev	CAACGGCCATTTAATTCTGG	This study
qNOT4 3'for	AACCGTCTCACAGCAAATCC	This study
qNOT4 3'rev	ACCGGCGATAATTTTCCTTC	This study
qIMP3prom for	CTATTTCCGCTCGCCTTTC	This study
qIMP3prom rev	TGTGATTATGCCTGGTGGT	This study
qIMP3orf for	TCAACGATCCAGCCTACCTC	This study
qIMP3orf rev	ATCGTCGATTTGGTTTCTGT	This study
qRNR1prom for	GGAGCTAATATTTCATTGTTGGAAA	This study
qRNR1prom rev	TGTTGTTCGTTGGTGTCTCTCT	This study
qRNR1ORF for	GCTCCAACAGGTAGCCACTC	This study
qRNR1ORF rev	AAATGTCGAACTCGGTTTCC	This study
qsnR9for	ATAGAACTTTCTACGCCTTTTCCTC	This study

qsnR9rev	GAAGGACTAATGATAGGTGGGTCA	This study
qU14for	TCACGGTGATGAAAGACTGG	This study
qU14rev	GGTCTCTAAAGAAGAGCGGTCA	This study
